# *PpMYB52* negatively regulates peach bud break through the gibberellin pathway and through interactions with *PpMIEL1*

**DOI:** 10.3389/fpls.2022.971482

**Published:** 2022-08-10

**Authors:** Yuzheng Zhang, Qiuping Tan, Ning Wang, Xiangguang Meng, Huajie He, Binbin Wen, Wei Xiao, Xiude Chen, Dongmei Li, Xiling Fu, Ling Li

**Affiliations:** ^1^College of Horticulture Science and Engineering, Shandong Agricultural University, Tai’an, China; ^2^State Key Laboratory of Crop Biology, Shandong Agricultural University, Tai’an, China; ^3^Shandong Collaborative Innovation Center for Fruit and Vegetable Production with High Quality and Efficiency, Shandong Agricultural University, Tai’an, China

**Keywords:** peach, bud break, *PpMYB52*, *PpMIEL1*, ABA, GA

## Abstract

Bud dormancy, which enables damage from cold temperatures to be avoided during winter and early spring, is an important adaptive mechanism of deciduous fruit trees to cope with seasonal environmental changes and temperate climates. Understanding the regulatory mechanism of bud break in fruit trees is highly important for the artificial control of bud break and the prevention of spring frost damage. However, the molecular mechanism underlying the involvement of MYB TFs during the bud break of peach is still unclear. In this study, we isolated and identified the *PpMYB52* (Prupe.5G240000.1) gene from peach; this gene is downregulated in the process of bud break, upregulated in response to ABA and downregulated in response to GA. Overexpression of *PpMYB52* suppresses the germination of transgenic tomato seeds. In addition, Y2H, Bimolecular fluorescence complementation (BiFC) assays verified that *PpMYB52* interacts with a RING-type E3 ubiquitin ligase, *PpMIEL1*, which is upregulated during bud break may positively regulate peach bud break by ubiquitination-mediated degradation of *PpMYB52*. Our findings are the first to characterize the molecular mechanisms underlying the involvement of MYB TFs in peach bud break, increasing awareness of dormancy-related molecules to avoid bud damage in perennial deciduous fruit trees.

## Introduction

Bud dormancy, which enables buds to avoid damage from cold temperatures during winter and early spring, is an important adaptive mechanism of deciduous fruit trees to cope with seasonal environmental changes and temperate climates (Yamane, 2014; Yordanov et al., 2014). The stages of bud dormancy is divided into paradormancy, endodormancy, and ecodormancy (Lang et al., 1987). Endodormancy is a complex mechanism regulated by multiple internal and external physiological factors and cannot released until certain chilling requirements are met (Singh et al., 2018). Ecodormancy is a growth stagnation caused by natural environmental, such as low temperature and drought (Horvath et al., 2003). With increasing global warming, fruit trees have been budding and blossoming earlier (Atauri et al., 2010). Trees that have broken bud are more vulnerable to late spring frost damage than trees yet to break bud (Jones and Cregg, 2006). Delaying bud break is one way to avoid spring frost damage (Chayani et al., 2015). Therefore, understanding the regulatory mechanism of bud break in fruit trees is highly important for the artificial control of bud break and the prevention of spring frost damage.

Bud dormancy is regulated by temperature, plant hormones, genes and other factors. ABA is generally considered to be a key hormone involved in dormancy induction and maintenance (Horvath et al., 2008; Gillespie and Volaire, 2017). And GA is considered to be a key hormone regulating bud dormancy release. The dormancy-related MADS-Box (DAM) Genes are Identified to be key factors controlling dormancy and dormancy release in many species, including leafy spurge (Horvath et al., 2010), pear (Niu et al., 2016), apple (Mimida et al., 2015), and peach (Hisayo et al., 2011). In addition to DAM, EARLY BUD BREAK 1 (*PpEBB1*) can promote bud break by regulating hormone metabolism, the cell cycle, and cell wall modifications (Zhao X. et al., 2020). *PpTCP20* can regulates peach flower bud endodormancy by negatively regulating the expression of *PpDAM5* and *PpDAM6*, and by interacting with *PpABF2* (Wang et al., 2020). Moreover, Gibberellin receptor GID1 gene might play a role in dormancy release in peach vegetative bud (Hollender et al., 2016).

MYBs constitute the largest transcription factor (TF) family in plants, and MYB TFs contain a highly conserved DNA-binding domain, the MYB domain, which typically contains one to four incomplete amino acid sequence repeats (R). Each R sequence consists of approximately 52 amino acids, forming three helices (Dubos et al., 2010). In addition, there are regularly spaced tryptophan (W) residues in each R sequence; these residues forms a hydrophobic core with a helix-turn-helix (HTH) structure between the second and third helices (Kanei-Ishii et al., 1990; Dubos et al., 2010).

The MYB TF family comprises proteins with a wide range of functions involved in the regulation of almost all biological processes in plants (Stracke et al., 2001; Dubos et al., 2010). A large number of studies have shown that plant MYB TFs are widely involved in the response to abiotic stress, such as drought (Huang et al., 2018; Alexander et al., 2019; Fang et al., 2019), heat (Li J. et al., 2021; Wu et al., 2021), cold (Gong et al., 2018), and salt (Zhao K. et al., 2020), and the regulation of the biosynthesis of various secondary metabolites, such as glucosinolates (Dubos et al., 2010), flavonoids (Kortstee et al., 2011; Anwar et al., 2019; Li Y. et al., 2020) and terpenoids (Matías-Hernández et al., 2017). In addition, MYB TFs are involved in the regulation of plant cell morphology and pattern developing (Lai et al., 2005; Guimil and Dunand, 2006) and the regulation of multiple growth and development processes (Byrne et al., 2000; Wang et al., 2009; Zhuang et al., 2021). In peach, the synthesis of anthocyanins and proanthocyanidins and the formation of trichomes on the surface of fruit are regulated by MYB TFs (Vendramin et al., 2014; Zhou et al., 2015; Rahim et al., 2019). In recent years, MYB TFs have been found to play an important role in regulating seed dormancy and germination. In Arabidopsis, *AtMYB96* inhibits seed germination by regulating the expression of abscisic acid (ABA)-insensitive 4 (ABI4) (Lee and Seo, 2015). *AtMYB7* can interact with *AtbZIP60* to coregulate seed germination (Xian et al., 2016). Moreover, the MYB TF family protein RSM1 can interact with HY5/HYH to regulate seed germination (Yang et al., 2018). In wheat, dormancy release was shown to occur earlier after the *TaMyB10-A1* gene was mutated (Mares and Himi, 2021). In maize, *ZmMYB59* plays a negative regulatory role in germination (Zhai et al., 2020). Studying tomato, Xu et al. (2018) found that overexpression of *SlMYB102* can improve the seed germination rate. In *Paeonia suffruticosa*, *PsMYB1* functions in response to low temperature to regulate bud dormancy release and germination (Zhang et al., 2015). However, there are few reports on MYB TFs regulating bud break in perennial woody plants, and the molecular mechanism underlying the involvement of MYB TFs in bud break remains unclear. In this study, we found that *PpMYB52*, which encodes a MYB TF in peach, was downregulated in the process of bud break, which may negatively regulate peach bud break.

To further explore the molecular mechanism of *PpMYB52* in peach bud break, a *PpMYB52-*interacting protein was identified from the peach dormancy-associated SSHcDNA library *via* a yeast two-hybrid (Y2H) assay, namely, RING-type E3 ligase MYB30-ligase 1 (*PpMIEL1*), a RING-type E3 ubiquitin ligase, which plays an important role in ubiquitin-mediated degradation of target proteins (Chen et al., 2021). RING-type E3 ubiquitin ligases compose a class of E3 ligases containing a RING-finger domain, and RING-type E3 ligases play an important role in abscisic acid signal transduction (Stone et al., 2006; Zhang et al., 2007); anthocyanin biosynthesis (An et al., 2017a,^2020^); and the response to abiotic stress, such as cold (An et al., 2020, 2021), drought (Bae et al., 2011) and salt (Du et al., 2021). In Arabidopsis, the ubiquitin ligase *AtMIEL1* mediates the degradation of *AtMYB30* and weakens plant defense (Marino et al., 2013). In addition, *AtMIEL1* also interacts with *AtMYB96* in Arabidopsis stems, and *AtMIEL1* can ubiquitinate *AtMYB96* and negatively regulate ABA sensitivity and cuticle wax biosynthesis (Lee and Seo, 2016; Lee et al., 2017). In apple, *MdMIEL1* can negatively regulate anthocyanin accumulation through ubiquitination-mediated degradation of *MdMYB1* proteins (An et al., 2017a) and interact with *MdMYB308L* to promote the ubiquitination-based degradation of *MdMYB308L*, which negatively regulates cold resistance and anthocyanin accumulation (An et al., 2020). However, there are few reports about MIEL1 in peach, and the molecular mechanism of *PpMIEL1* in bud break is still unclear.

In this study, we found that peach *PpMYB52*, which is upregulated by ABA and downregulated by gibberellins (GAs), negatively regulates peach bud break. In addition, we identified a RING-type E3 ubiquitin ligase, *PpMIEL1*, which interacts with *PpMYB52*, and its expression was continuously upregulated during bud break. We hypothesized that *PpMIEL1* may positively regulate peach bud break by the ubiquitination and degradation of *PpMYB52*.

## Materials and methods

### Plant materials and treatments

Peach (*Prunus persica* var. *nectarina* cv. Zhongyou 4) trees were grown at the Shandong Agricultural University Horticultural Experiment Station in Tai’an, Shandong Province. Flower bud samples were collected approximately every 15 days from 15 October 2021 to 15 March 2022.Phloem samples were taken from the middle and lower part of annual branches. Two ring cuts of the branches with an interval of 5cm and two longitudinal cuts on both sides of the branches were cut with a knife. All cuts were down to the xylem. The phloem was gently raised with the knife edge and then quickly placed in liquid nitrogen.

Eighteen annual shoots with a length of 40–50 cm were removed from Zhongyou 4 described above, and were divided into three groups, namely, those treated with deionized water + 0.5% Triton 100, 1 mM GA_3_ + 0.5% Triton 100 and 1 mM ABA + 0.5% Triton 100, ABA and GA_3_ were dissolved in 8 mL ethanol, thendeionized water and 2.5 ml Triton 100 were added to 500 ml, the same volume of ethanol and Triton 100 were added to the thendeionized water, and the solution was evenly sprayed onto the branches, on 30 January. The shoots of each treatment group were placed in tap water under 200 μmol m^–2^ s^–1^ light for 16 h at 25°C and 8 h of darkness at 23°C and were then collected at 0, 1, 5, 9, and 14 days after treatment. All the flower buds were removed, immediately placed in liquid nitrogen and then stored at -80°C for subsequent experiments.

To determine the flower bud break rate of cultivars throughout the dormancy period, at each sampling time, 25 shoots were placed in tap water under 200 μmol m^–2^ s^–1^ light for 16 h at 25°C and 8 h of darkness at 23°C with a relative humidity of 75%. After 25 days, the percentage of flower buds that had broken dormancy was determined. If the bud break was less than 50%, the flower buds were considered to be in the endodormancy stage (Lang, 1987).

### RNA isolation and quantitative PCR

Total RNA was extracted from 0.1 g of peach bud tissue and tomato leaves using an RNAprep Pure Plant Kit (Tiangen, Beijing, China) according to the manufacturer’s instructions. First-strand cDNA was then generated using HiScript qRT SuperMix for qPCR (+ gDNA-wiper) (Vazyme, Nanjing, China) according to the manufacturer’s instructions. Quantitative PCR (qPCR) was performed on a CFX96 real-time PCR detection system (Bio-Rad) together with SYBR premix Ex Taq (Takara). Three biological replicates were included for each analysis. The relative expression levels were calculated using the 2^–Δ^ ^Δ^ *^CT^* method (Livak and Schmittgen, 2001), with the *PpUBQ* gene used as an internal control in peach and the SlActin gene used as an internal control in tomato. The primers used are shown in Supplementary Table 1. The data were analyzed using SPSS Statistics v20 to analyze the significance of the differences among data, with a significance level of *p* < 0.05 under Duncan’s test.

### Vector construction and genetic transformation

The full-length open reading frames (ORFs) of *PpMYB52* and *PpMIEl1* were amplified using flower bud cDNA *via* 2 × Phanta Max Master Mix (P515, Vazyme) according to the manufacturer’s instructions. The primers used were designed using CE Design v1.04 (Vazyme) and are shown in Supplementary Table 1. The vectors were constructed using a ClonExpress Ultra One Step Cloning Kit (C115, Vazyme) according to the manufacturer’s instructions. Full-length *PpMYB52* was inserted into the pRI101 vector under the control of the CaMV35S promoter. Subsequently, the obtained 35S:*PpMYB52* vector was transformed into *Agrobacterium tumefaciens* strain GV3101 according to the freeze–thaw method, which was then used to infect Micro Tom tomato explants (Jian et al., 2019). DNA was extracted for PCR for transgene detection. Three independent homozygous T3 transgenic lines (OE-1, OE-2, and OE-3) were selected for subsequent experiments.

### Yeast two-hybrid assays

**FIGURE 1:**
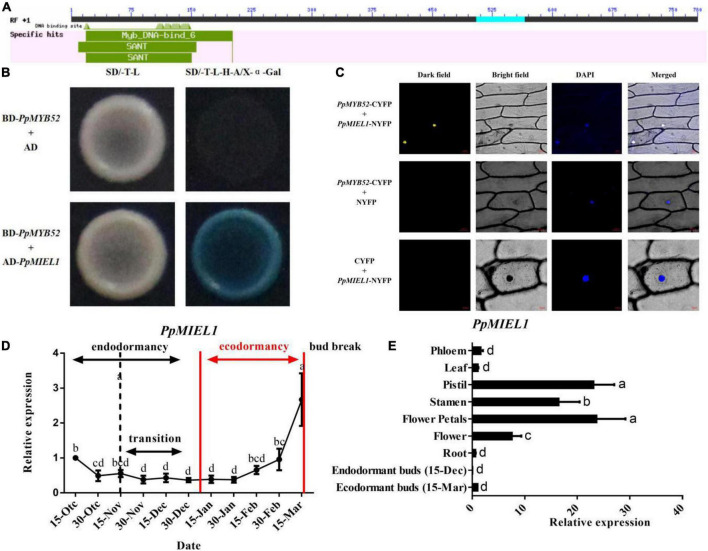
*PpMYB52* interaction with *PpMIEL1* and the expression pattern of *PpMIEL1*. **(A)** Analysis results of conserved domains according to the NCBI. **(B)** *PpMYB52* interacts with *PpMIEL1* in yeast. Because of the autoactivation of the full-length *PpMYB52* protein, we used a 1–300 bp region to perform the experiment. Yeast cells transformed with BD-*PpMYB52* (1–300 bp) + AD were included as negative controls. **(C)** *PpMYB52* and *PpMIEL1* BiFC verification. **(D)** Expression level of PpMIEL1 during the various stages of dormancy and bud break from 15 October 2021 to 15 March 2022. **(E)** Expression of *PpMIEL1* in different tissues of Zhongyou 4 peach.

A peach dormancy-associated SSHcDNA library was constructed from peach buds collected from dormancy until bud break. The full-length ORF, 1–300 bp ORF and 301–777 bp ORF of *PpMYB52* were inserted into a pGBKT7 bait vector for verification of self-activation, the 1–300 bp region of *PpMYB52* was used to screen interacting genes whose products contain an Myb_DNA-bind_6 domain, as the full-length *PpMYB52* protein had self-activation activity (Figure 1A and Supplementary Figure 1). And the full-length ORF of *PpMIEl1* was cloned into a pGADT7 vector *via* the primers listed in Supplementary Table 1. The two recombinant plasmids were cotransformed into yeast Y2H Gold strains, and the transformants were cultured on selective media (SD/-Trp/-Leu) at 30°C for ∼3 days. After the yeast cells had grown, the putative transformants (OD600 = 0.002) were transferred to selective media (SD/-Ade/-His/-Leu/-Trp/x-α-Gal).

### Bimolecular fluorescence complementation assays

The full-length ORF of *PpMYB52* was cloned into a pSPYNE vector, yielding a *PpMYB52*-NYFP plasmid, and the full-length ORFs of *PpMIEl1* were inserted into a pSPYCE vector, yielding a *PpMIEl1*-CYFP plasmid. All the recombinant plasmids were individually transformed into *A. tumefaciens* strain GV3101. Equal concentrations of *A. tumefaciens* strain GV3101 containing the plasmids of interest were transiently coexpressed in onion epidermal cells. After incubation at 25°C in the dark for 48 h, fluorescent and differential interference contrast (DIC) images were observed with a laser-scanning confocal microscope (Zeiss LSM880), and the images were analyzed using Zen Lite software (Zeiss) and Adobe Photoshop 7.0. The yellow fluorescent protein (YFP) was visualized by excitation with an argon laser at 514 nm.

### Subcellular localization of *PpMYB52*

The ORF sequence of *PpMYB52* without the stop codon was amplified and ligated into a pRI101-GFP (35S:GFP) vector for detection of subcellular localization (Hu et al., 2016). The primers used are listed in Supplementary Table 1. *PpMYB52*-GFP and a control GFP construct were infiltrated into 4-week-old tobacco (*Nicotiana benthamiana*) leaves *via A. tumefaciens* strain GV3101. After 3 days of incubation, the GFP fluorescence signals in the transformed onion cells were observed using a Zeiss LSM880 microscope, images were collected, and the images were analyzed using Zen Lite software (Zeiss).

### Germination of transgenic tomato seeds

Seeds from three homozygous T3 transgenic tomato lines overexpressing *PpMYB52* were surface disinfected with 75% ethanol for 60 s followed by 50% sodium hypochlorite for 15 min and then washed with sterile water five times (5 min each time). Finally, the seeds were cultured on Murashige and Skoog (MS) solid media, and the germination was recorded every 12 h.

## Results

### The transcript level of *PpMYB52* is negatively correlated with peach flower bud break

**FIGURE 2:**
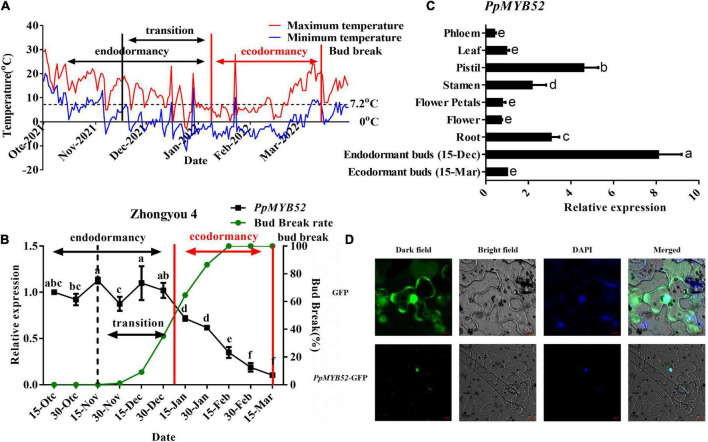
Definition of the dormancy stages of Zhongyou 4 flower buds and expression patterns of *PpMYB52*. **(A)** Daily maximum temperature and daily minimum temperature from October 2021 to March 2022. **(B)** Definition of the dormancy stages of Zhongyou 4 flower buds and expression level of *PpMYB52* from 15 October 2021 to 15 March 2022. At each sampling time, 25 of the shoots were placed in tap water for 25 days. If less than 50% of the buds broke dormancy, the flower buds were considered to be in the endodormancy stage; the values are the means of 25 shoots. **(C)** Expression of *PpMYB52* in different tissues of Zhongyou 4 peach. **(D)** Subcellular localization of *PpMYB52*. PpMYB52 was fused to YFP for transient transformation into 4-week-old tobacco (*N. benthamiana*) leaves. 4’,6-Diamidino-2-phenylindole (DAPI) was used as a nuclear marker. The values represent the means ± *SD* of three replicates, and the different letters above the bars represent significant differences; *P* < 0.05.

To evaluated the relative expression of *PpMYB52* from dormancy to bud break, we first identified the dormancy stages of Zhongyou 4 flower buds. The endodormancy stage lasted from 15 October to 30 December, and the flower buds broke in the field a few days after sampling on March 15 (Figure 2B). The expression of *PpMYB52* was maintained at a high level in the endodormancy stage, continued to decrease in the process of bud break (the period from endodormancy to bud break, which is also known as ecodormancy), and decreased to the lowest level before bud break (Figure 2B). And the temperature during the whole dormancy process is shown in Figure 2A. Tissue-specific analysis showed that the *PpMYB52* gene was highly expressed in peach buds during endodormancy but was expressed at low levels in the flower buds before bud break, and in the flowers, petals, leaves and phloem (Figure 2C). Subcellular localization revealed that the *PpMYB52* gene was localized in the nucleus (Figure 2D).

### PpMYB52 transcription is regulated by gibberellin and abscisic acid

**FIGURE 3:**
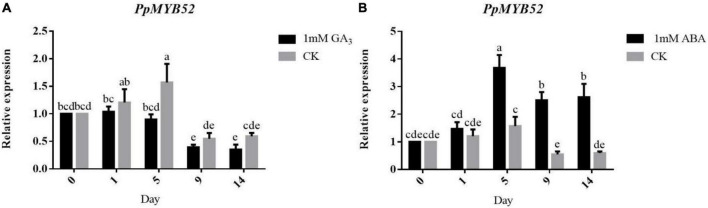
Expression of *PpMYB52* in the flower buds of Zhongyou 4 under GA_3_ and ABA pretreatments. **(A)** Expression of *PpMYB52* in the flower buds of Zhongyou 4 under 1 mM GA_3_ + 0.5% Triton 100 treatment. **(B)** Expression of *PpMYB52* in the flower buds of Zhongyou 4 under 1 mM ABA + 0.5% Triton 100 treatment. Deionized water + 0.5% Triton 100 was used as a blank control (CK). The values represent the means ± *SD* of three replicates, and the different letters above the bars represent significant differences; *P* < 0.05.

GA and ABA have been recognized as key internal factors of dormancy and bud break. To determine the response of *PpMYB52* to GA and ABA, we treated the shoots of Zhongyou 4 with 1 mM GA_3_ and 1 mM ABA, and deionized water was used as a blank control. The results showed that, compared with that in peach flower buds treated with deionized water, the expression of *PpMYB52* in peach flower buds treated with 1 mM GA_3_ was downregulated (Figure 3A). In contrast, the expression of *PpMYB52* in peach flower buds treated with 1 mM ABA was upregulated (Figure 3B), and the upregulation was more obvious with the extension of treatment time.

### Overexpression of *PpMYB52* suppresses germination and vegetative growth of transgenic tomato

**FIGURE 4:**
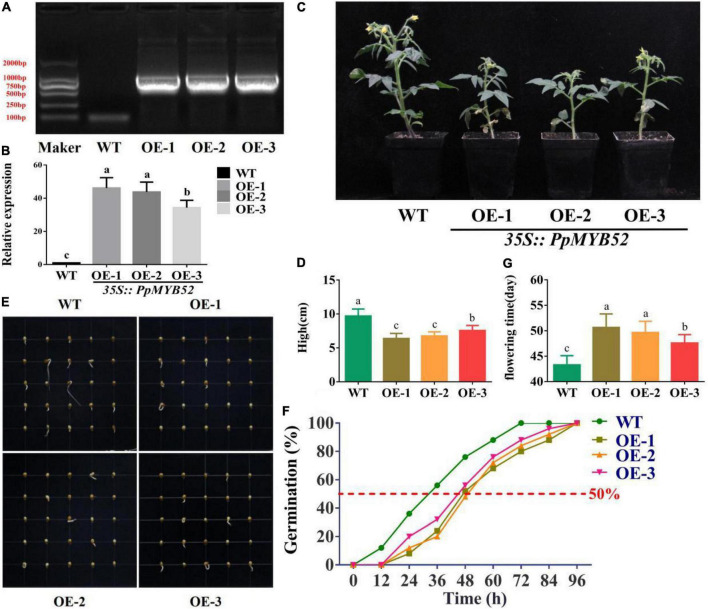
Overexpression of *PpMYB52* in tomato suppresses seed germination and reduces plant height. **(A)** Reverse transcription polymerase chain reaction (RT-PCR) analysis results of *PpMYB52*-overexpressing transgenic lines. **(B)** qRT-PCR analysis results of *PpMYB52* transgenic tomato lines under normal conditions. *SlActin* was used as a reference gene. **(C)** Phenotypes of *PpMYB52-*overexpressing transgenic tomato and Micro Tom wild-type tomato. **(D)** Plant height of *PpMYB52-*overexpressing transgenic tomato and Micro Tom wild-type tomato. Each group comprised 20 plants, and the results are the averages of 20 plants. **(E,F)** Determination of the germination of seeds of Micro Tom wild-type tomato and *PpMYB52*-overexpressing transgenic tomato in MS media. **(G)** Flowering time of *PpMYB52*-overexpressing transgenic tomato and Micro Tom wild-type tomato. The time is the number of days between planting and the first flower opening. Each group comprised 20 plants, and the results are the averages of 20 plants. The values represent the means ± *SD* of three replicates, and the different letters above the bars represent significant differences; *P* < 0.05.

To elucidate the function of *PpMYB52* in bud break, a 35S:*PpMYB52* fusion plasmid was heterologously transformed into tomato; tomato was used instead of peach because transgenic peach plants are difficult to obtain. We obtained three independent transgenic lines (OE-1, OE-2, and OE-3), which were identified *via* PCR and qRT–PCR (Figures 4A,B). The plant height of *PpMYB52* overexpression transgenic tomato lines was lower than that of the wild-type plants (Figures 4C,D). The flowering time of *PpMYB52* overexpression transgenic tomato lines was longer than that of the wild-type plants (Figures 4C,G). To determine the function of *PpMYB52* in bud break, the seeds of the three homozygous T3 *PpMYB52*-overexpressing transgenic line and Micro Tom wild-type tomato plants were sown onto MS media, and the germination was observed. The results showed that it took a longer time for the transgenic lines to achieve the same germination as that of the wild-type tomato and the transgenic lines took longer to reach 50% germination than the wild type tomato (Figures 4E,F), which indicated that the overexpression of *PpMYB52* inhibited the germination of transgenic tomato seeds.

### Overexpression of *PpMYB52* inhibits the expression of key genes involved in gibberellin synthesis and promote the expression of key genes involved in gibberellin deactivated of transgenic tomato

**FIGURE 5:**
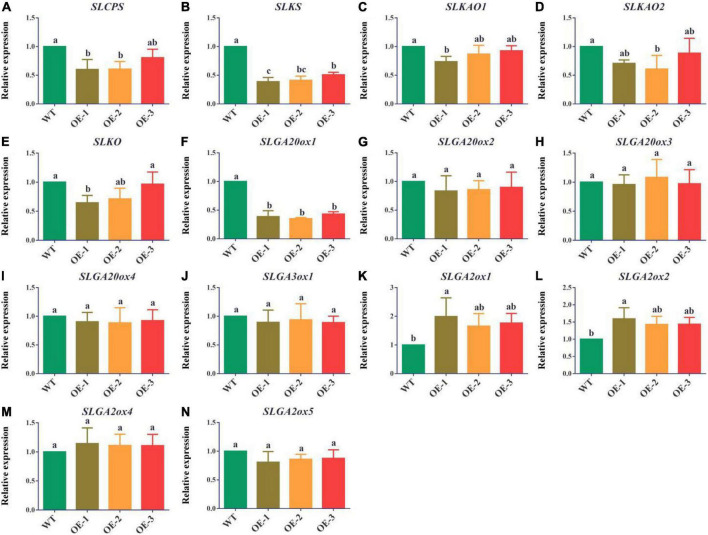
Expression of GA biosynthesis genes and GA deactivated genes in *PpMYB52*-overexpressing transgenic tomato and Micro Tom wild-type tomato. **(A)** Expression of *SLCPS*. **(B)** Expression of *SLKS*. **(C)** Expression of *SLKAO1*. **(D)** Expression of *SLKAO2*. **(E)** Expression of *SLKO*. **(F)** Expression of *SLGA20ox1*. **(G)** Expression of *SLGA20ox2*. **(H)** Expression of *SLGA20ox3*. **(I)** Expression of *SLGA20ox4*. **(J)** Expression of *SLGA3ox1*. **(K)** Expression of *SLGA2ox1*. **(L)** Expression of *SLGA2ox2*. **(M)** Expression of *SLGA2ox4*. **(N)** Expression of *SLGA2ox5*. The values represent the means ± *SD* of three replicates, and the different letters above the bars represent significant differences; *P* < 0.05.

GAs are generally considered to be key hormones involved in regulating bud break. To determine the effect of *PpMYB52* on GA synthesis, we measured the expression of genes encoding key enzymes in GA synthesis in both transgenic lines and wild-type plants. The results showed that the expression of GA biosynthesis genes copalyl diphosphate synthase (*SLCPS*), ent-kaurene synthase (*SLKS*), ent-kaurenoic acid oxidase1 (*SLKAO1*), ent-kaurenoic acid oxidase2 (*SLKAO2*) and gibberellin 20-oxidase-1(*SLGA20ox1*) in the *PpMYB52*-overexpressing transgenic tomato were lower than that in Micro Tom wild-type tomato (Figures 5A–D,F). The expression of GA deactivated genes gibberellin 2 -oxidase-1 (*SLGA2ox1*) and gibberellin 2 -oxidase-2(*SLGA2ox2*) in the *PpMYB52*-overexpressing transgenic tomato were higher than that in Micro Tom wild-type tomato (Figures 5K,L). However, the expression of *SLKO*, *SLGA20ox2*, *SLGA20ox3*, *SLGA20ox4*, *SLGA3ox1*, *SLGA2ox4*, and *SLGA2ox5* showed no significant difference between transgentic tomato and wild-type tomato (Figures 5E,G–J,M,N). Taken together, these results suggest that overexpression of *PpMYB52* inhibits GA synthesis and promote GA deactivation.

### *PpMYB52* interacts with *PpMIEL1*

To further explore the regulatory mechanism of *PpMYB52*, a number of proteins that may interact with *PpMYB52* were screened from the peach dormancy-associated SSHcDNA library (Supplementary Table 2). Five genes (Supplementary Table 2) were screened and subsequently tested for their ability to interact with *PpMYB52*. The full-length cDNA of these genes was inserted into a pGADT7 vector as prey, and BD-*PpMYB52* (1–300 bp) was used for transformation into yeast receptor cells in pairs for point-to-point verification. The RING-type E3 ligase MYB30-INTERACTING E3 LIGASE 1 (*PpMIEL1*; Prupe.1G141000) gene was found to interact with the Myb_DNA-bind_6 domain (1–300 bp) of PpMYB52 (Figure 1B), which might function in ubiquitination-mediated degradation of target proteins. We performed a BiFC assay to confirm the interaction between *PpMYB52* and the *PpMIEL1* protein *in vivo*. *PpMYB52* was fused to the N-terminus of enhanced YFP (NYFP), and *PpMIEL1* was fused to the C-terminus of enhanced YFP (CYFP). These constructs were subsequently transformed into onion epidermal cells and expressed transiently. Nuclear fluorescence was detected when *PpMYB52* was coexpressed with *PpMIEL1*, but in the control experiments, no YFP fluorescence was detected. Taken together, these results indicate that *PpMYB52* interacts with the *PpMIEL1* protein *in vivo* (Figure 1C).

### *PpMIEL1* may positively regulate peach flower bud break

The expression of *PpMIEL1* was maintained at a low level in the endodormancy stage, increased in the process of bud break, and decreased to the highest level before bud break (Figure 1D), which contrasts with the expression of *PpMYB52* (Figure 2B). Tissue-specific analysis showed that the *PpMIEL* gene was highly expressed in the flower petals, stamens, and pistils and was expressed the lowest in the peach buds during endodormancy (Figure 1E), which is quite different from the results of *PpMYB52*. Overall, these results showed that *PpMIEL1* may positively regulate peach flower bud break.

## Discussion

### *PpMYB52* is a negative regulator of bud break in peach

In previous studies, MYB TFs have been found to be closely related to seed germination. Specifically, *AtMYB44*, *AtMYB96*, and *ZmMYB59* play a negative regulatory role in seed germination (Nguyen et al., 2012; Lee and Seo, 2015; Zhai et al., 2020). In this study, we found that the expression of *PpMYB52* was maintained at a high level in the endodormancy stage, continued decrease throughout bud break, and decreased to the lowest level before bud break (Figure 2B). Further research showed that the overexpression of *PpMYB52* inhibited the germination of transgenic tomato seeds (Figures 4E,F). These results suggest that *PpMYB52* is a negative regulator of bud break in peach.

The role of GA in promoting dormancy release and bud break has been widely studied (Gillespie and Volaire, 2017). GA_3_ treatment has been shown to be a good method for breaking bud dormancy in several tree species (Zhuang et al., 2015). In poplar, the *GA2ox4* gene, which is downregulated during temporal events that lead to poplar bud break, is involved in GA catabolism (Gómez-Soto et al., 2021). In *Salix pentandra*, long-day induced bud break is associated with transiently increased levels of GA_1_ (Olsen et al., 1997). In Japanese apricot, exogenous GA_4_ promotes flower bud break (Zhuang et al., 2015). In peach, GA_3_, GA_4_, and GA_5_ promote dormancy release (Li S. et al., 2021). In chrysanthemum, Zhu et al. (2020) found that *CmMYB2* interacts with *CmBBX24* to regulate flowering by influencing GA synthesis. In this study, overexpression of *PpMYB52* inhibited the expression of *SLCPS*, *SLKS, SLKAO1, SLKAO2*, and *SLGA20ox1* in tomato (Figures 5A–D,F) and promote the expression of *SLGA2ox1* and *SLGA2ox2* (Figures 5K,L). CPS, KS, KAO and GA20ox are key enzymes in the biosynthesis of GAs, and GA2ox is key enzyme in the deactivation of GAs (Hayashi et al., 2006; Yamaguchi, 2006; Huang et al., 2012). In addition, the height of the *PpMYB52*-overexpressing transgenic tomato lines was lower than that of the wild type (Figures 4C,D), which might mean that the *PpMYB52*-overexpressing transgenic tomato lines had lower GA contents. These results suggest that overexpression of *PpMYB52* might inhibit the germination of transgenic tomato seeds by inhibiting GA synthesis and promote GA deactivation.

### Gibberellin and abscisic acid regulate the expression of *PpMYB52*

Numerous studies have shown that plant hormones, especially ABA and GAs, play important roles in regulating dormancy and bud break (Horvath et al., 2008; Aksenova et al., 2013; Gillespie and Volaire, 2017). The role of GA in regulating dormancy release and bud break is described above. ABA is generally considered to be a key hormone involved in regulating bud dormancy (Cooke et al., 2012; Vergara et al., 2017). Moreover, ABA can delay the germination of seeds until more suitable growing conditions occur, increasing survival (Richardson et al., 2019). Continued *in situ* ABA biosynthesis is required for the maintenance of bud dormancy (Le Bris et al., 1999).

MYB TFs have been found to respond to plant hormone signals and are widely involved in hormone-regulated plant growth and development (Jaradat et al., 2013; Yin et al., 2020). In rice, the *OsGAmyb* gene was shown to be induced in response to GA signaling and participates in seed germination (Gubler et al., 1997). In addition, by interacting with SLENDER RICE 1 (SLR1), a DELLA repressor of GA signaling, *OsMYB103L* is involved in GA-mediated regulation of the cellulose synthesis pathway (Ye et al., 2015). In *Lolium temulentum*, *LtGAMYB* is upregulated in response to GA_3_ in the seed and participates in the flowering process (Gocal et al., 1999). In this study, we found that *PpMYB52* can also respond to GA signaling and that exogenous GA_3_ treatment can inhibit the expression of *PpMYB52* (Figure 3A). In addition to GA signaling, many studies have found that MYB TFs can also respond to ABA signaling. In Arabidopsis, *AtMYB30* and *AtMYB52* are involved in the ABA response (Park et al., 2011; Zheng et al., 2012). In grapevine, the expression of *VvMYB60* increases in response to ABA (Galbiati et al., 2011). In this study, we found that *PpMYB52* can also respond to ABA signaling and that exogenous ABA treatment can promote the expression of *PpMYB52* (Figure 3B). Endogenous GA levels increase during the onset of bud break, and the ABA concentration decreases with dormancy progression (Gillespie and Volaire, 2017). Therefore, we hypothesized that the continuous downregulation of *PpMYB52* from endodormancy to bud break might be regulated by ABA and GA.

### *PpMIEL1* may positively regulate peach flower bud break by interacting with *PpMYB52*

*MIEL1* is a RING-type E3 ubiquitin ligase that plays an important role in ubiquitin-mediated degradation of target proteins (Chen et al., 2021). In this study, we used Y2H assays to identify the interacting protein of *PpMYB52*, namely, the RING-type E3 ubiquitin ligase *PpMIEL1*, which was confirmed by BiFC analysis (Figures 1B,C). An et al. (2017b) found that ectopic expression of *MdMIEL1* in Arabidopsis produced early-germinating phenotypes relative to those of wild-type plants. In this study, we found that *PpMIEL1* expression was maintained at a low level in the endodormancy stage, increased during bud break, and decreased to the highest level before bud break (Figure 1D), which was quite different from the pattern of *PpMYB52* (Figure 2B). These results showed that *PpMIEL1* may positively regulate peach flower bud break. Therefore, we speculate that the expression of *PpMIEL1* increased during the bud break process in peach flower buds, which interact with *PpMYB52* and may promoting its ubiquitination-mediated degradation and bud break.

## Conclusion

**FIGURE 6:**
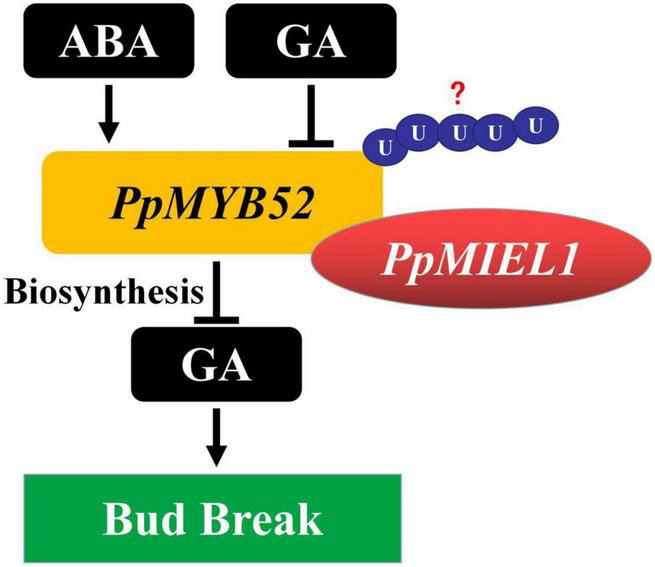
Model for *PpMYB52* regulation of bud break in peach flower buds. *PpMYB52* is upregulated by ABA and downregulated by GA, which negatively regulates peach bud break by inhibiting GA synthesis. And *PpMYB52* interacts with *PpMIEL1*, which is upregulated in the process of bud break and may positively regulate peach bud break by ubiquitination-mediated degradation of *PpMYB52*.

In summary, the results of our study demonstrate that *PpMYB52*, which is upregulated by ABA and downregulated by GA, negatively regulates peach bud break. In addition, we found that *PpMYB52* interacts with a RING-type E3 ubiquitin ligase, *PpMIEL1*, which is upregulated in the process of bud break and may positively regulate peach bud break by ubiquitination-mediated degradation of *PpMYB52* (Figure 6). This research characterized a potential mechanism of the involvement of MYB TFs in peach bud break, highlighting a new way for dormancy-related molecules to avoid bud damage in perennial deciduous fruit trees.

## Data availability statement

The original contributions presented in this study are included in the article/Supplementary material, further inquiries can be directed to the corresponding author/s.

## Author contributions

LL, XF, and YZ designed the study. YZ, QT, NW, XM, HH, BW, WX, XC, and DL performed the experiments and analyzed the data. YZ wrote the manuscript. All authors contributed to the article and approved the submitted version.

## Abbreviations

ABA: abscisic acid
BiFC: bimolecular fluorescence complementation
cDNA: complementary DNA
CDS: coding sequence
DNA: desoxyribose nucleic acid
GFP: green fluorescent protein
YFP: yellow fluorescent protein
MS: murashige and skoog medium
OD: optical density
ORF: open reading frame
qRT-PCR: real-time quantitative
RNA: ribonucleic acid
X-α-gal: 5-bromo-4-chloro-3-indolyl-α-D-galactoside
Y2H: yeast two-hybrid.
