# Supplementary material for: PpMYB52 negatively regulates peach bud break through the gibberellin pathway and through interactions with PpMIEL1
Source: Front Plant Sci. 2022 Aug 10;13:971482. doi: 10.3389/fpls.2022.971482 (PMC9413399; doi:10.3389/fpls.2022.971482)
Supplement: Supplementary file 1 [file Data_Sheet_1.docx]

Appendix A. Supplementary Data


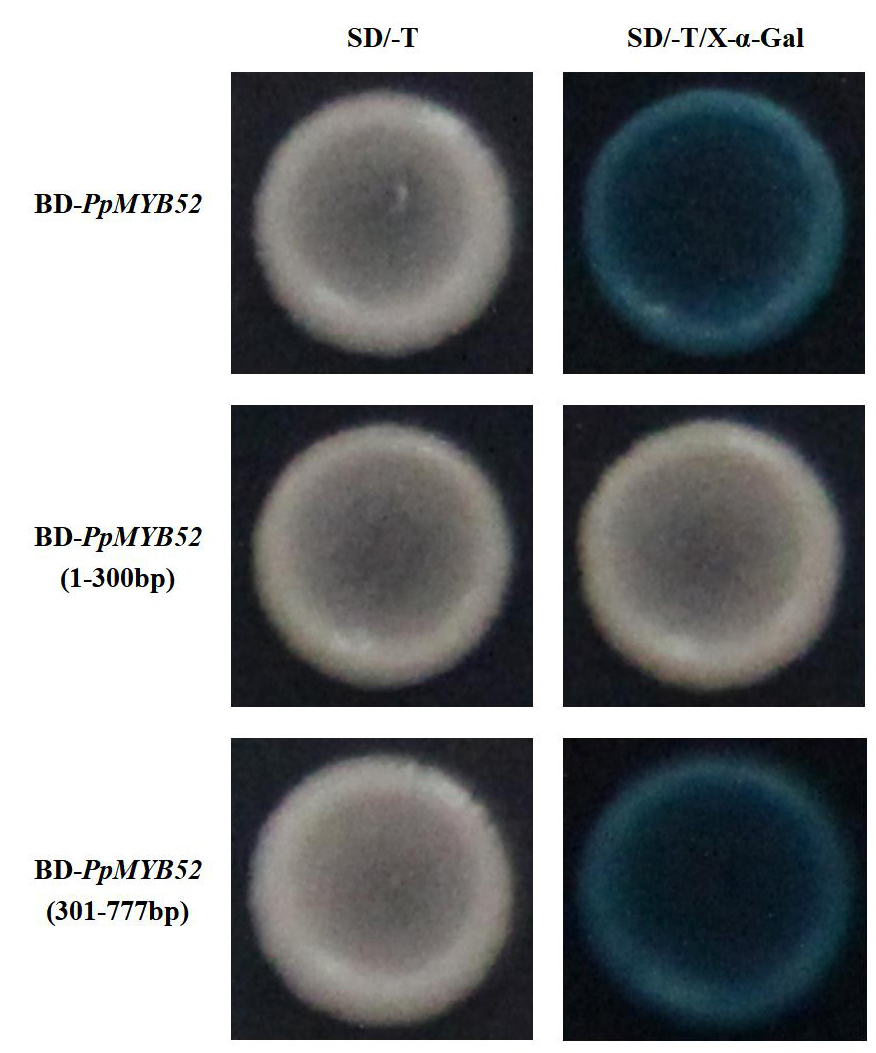


**Supplementary** **Figure 1.** Identification of *PpMYB52* self-activation.

**Supplementary Table 1.** Primers used in this [research](file:///E:\\javascript:;).

| Primer name | Primer sequence (5'→3') | Usage |
| --- | --- | --- |
| BD-PpMYB52-F | tcagaggaggacctgcatatgATGTGCACTAGAGGCCACTGG | Y2H |
| BD-PpMYB52-R | tcgacggatccccgggaattcAGAACTTCCATTAACAGACAAGAACTCT | Y2H |
| BD-PpMYB52 (1-300 bp)-F | tcagaggaggacctgcatatgATGTGCACTAGAGGCCACTGG | Y2H |
| BD-PpMYB52 (1-300 bp)-R | tcgacggatccccgggaattcGATGACATGCCAATGGTTCTTCA | Y2H |
| BD-PpMYB52 (301-777 bp)-F | tcagaggaggacctgcatatgATGGCACGCAGATGCAGAG | Y2H |
| BD-PpMYB52 (301-777 bp)-R | tcgacggatccccgggaattcAGAACTTCCATTAACAGACAAGAACTCT | Y2H |
| AD-PpMIEL1-F | gtaccagattacgctcatatgATGGGAGGCTCAGGGAATGA | Y2H |
| AD-PpMIEL1-R | atgcccacccgggtggaattcTTGAGGAAGAACTGGGGGGG | Y2H |
| 5BD | GTGCGACATCATCATCGGAAG | Y2H |
| 3BD | TAAGAGTCACTTTAAAATTTGTATAC | Y2H |
| 5AD | AATACCACTACAATGGATGATG | Y2H |
| 3AD | GAGATGGTGCACGATGCACAGT | Y2H |
| PBI121(35S) | GACGCACAATCCCACTATCC | Transgene and subcellular localization |
| PpMYB52-GFP-F | ttgatacatatgcccgtcgacATGTGCACTAGAGGCCACTGG | Transgene and subcellular localization |
| PpMYB52-GFP-R | tttacccatgaattcggatccAGAACTTCCATTAACAGACAAGAACTCT | Transgene and subcellular localization |
| PpMYB52-CYEP-F | tggcgcgccactagtggatccATGTGCACTAGAGGCCACTGG | BiFC |
| PpMYB52-CYEP-R | agcggtaccctcgaggtcgacAGAACTTCCATTAACAGACAAGAACTCT | BiFC |
| PpMIEL1-NYEP-F | tggcgcgccactagtggatccATGGGAGGCTCAGGGAATGA | BiFC |
| PpMIEL1-NYEP-R | agcggtaccctcgaggtcgacTTGAGGAAGAACTGGGGGGG | BiFC |
| RT-PpMYB52-F | GCTTAGGGAGTTGGTGGAA | qRT–PCR |
| RT-PpMYB52-R | TGGATACGGTGAGAGGCTA | qRT–PCR |
| RT-PpMIEL1-F | GAAGTGTGGGTCTTGCTATTC | qRT–PCR |
| RT-PpMIEL1-R | TCTATTCTCCTCCAGGTTCTG | qRT–PCR |
| RT-SLMYB52-F | CCAGGTAGAACTGATAATGCTG | qRT–PCR |
| RT-SLMYB52-R | CTCCTCTTCCGTGACTTGA | qRT–PCR |
| RT-SLCPS-F | AGATTTAGCCTCACTGCTCAC | qRT–PCR |
| RT-SLCPS-R | GATGTCGTCCCACTTGATAAC | qRT–PCR |
| RT-SLKS-F | GGTTTGAGTTTCACCCAGC | qRT–PCR |
| RT-SLKS-R | CCCAGGCAGTATCGTATGA | qRT–PCR |
| RT-SLKAO1-F | TCTCCTTTGTGGTCTTTCG | qRT–PCR |
| RT-SLKAO1-R | GGTGTAAGTCCATCCCATCT | qRT–PCR |
| RT-SLKAO2-F | AAGGTATGGACAAGGTGGAA | qRT–PCR |
| RT-SLKAO2-R | CGCTTGTGTTCTTGGCTT | qRT–PCR |
| RT-SLKO-F | ATTGGGCTGAAACTTATGGACC | qRT–PCR |
| RT-SLKO-R | CGCTTCGCTGTCTTGTGAA | qRT–PCR |
| RT-SLGA20ox1-F | GAGAAGCCTTGTGCCTTAGT | qRT–PCR |
| RT-SLGA20ox1-R | GATTAGCATCAACTCCGTGA | qRT–PCR |
| RT-SLGA20ox2-F | TTGTAATGCGATGAGCACTC | qRT–PCR |
| RT-SLGA20ox2-R | TGTGGTCCAGTTCCTAACG | qRT–PCR |
| RT-SLGA20ox3-F | CGTGAGCACCCTTTCTCTA | qRT–PCR |
| RT-SLGA20ox3-R | GCCCACCAACACTATCTTG | qRT–PCR |
| RT-SLGA20ox4-F | TTGAATGGCTATCTCTCCG | qRT–PCR |
| RT-SLGA20ox4-R | ACAAGTGGCTTCCCAAAG | qRT–PCR |
| RT-SLGA3ox1-F | AGGGTTCACCATTGTTGG | qRT–PCR |
| RT-SLGA3ox1-R | TTGTAAGGCAGCACATCCT | qRT–PCR |
| RT-SLGA2ox1-F | AAGGGTCCAACAAACTTCAG | qRT–PCR |
| RT-SLGA2ox1-R | CTGTATGCTCTCCAAATCCA | qRT–PCR |
| RT-SLGA2ox2-F | TTGACCTCTCTAAACCCGAC | qRT–PCR |
| RT-SLGA2ox2-R | GACCACAATCGCCATTTG | qRT–PCR |
| RT-SLGA2ox4-F | CTGCCATTACTCACTTCCCT | qRT–PCR |
| RT-SLGA2ox4-R | CCTGCCTTTAGTTTCTCAGAG | qRT–PCR |
| RT-SLGA2ox5-F | GACCTCTCTAAACCTGACTCCA | qRT–PCR |
| RT-SLGA2ox5-R | TATCGCCACTTTGTCCGA | qRT–PCR |
| UBQ-F | CGAACCCTAGCCGATTACAA | Actin |
| UBQ-R | AGTGGTTCGCCATGAAAGTC | Actin |
| SlAction -F | GTCCTCTTCCAGCCATCCA | Actin |
| SlAction -R | ACCACTGAGCACAATGTTACCG | Actin |

**Supplementary Table 2.** Genes that were screened from the peach dormancy-associated SSHcDNA library.

| Gene ID: | Annotate: |
| --- | --- |
| Prupe.1G141000* | RING-type E3 ligase MYB30-INTERACTING E3 LIGASE 1 |
| Prupe.5G099200* | PROTEIN KINASE PROTEIN WITH TETRATRICOPEPTIDE REPEAT DOMAIN |
| Prupe.8G182300 | Hydroxymethylglutaryl-CoA reductase (NADPH)/HMG-CoA reductase |
| Prupe.4G288000 | CHLORIDE CHANNEL PROTEIN CLC-C |
| Prupe.1G290600 | SRF-type transcription factor (DNA-binding and dimerization domain) (SRF-TF) |
| Prupe.5G123200* | SF38-COPINE |
| Prupe.2G262000 | GANGLIOSIDE INDUCED DIFFERENTIATION ASSOCIATED PROTEIN 2-RELATED |
| Prupe.5G018200 | Transferase family (Transferase) |
| Prupe.6G016700 | D-mannose binding lectin (B_lectin) |
| Prupe.5G145000 | Uncharacterized conserved protein |
| Prupe.7G246800* | ACTIN-RELATED PROTEIN 6 |
| Prupe.5G006500 | VACUOLAR ATP SYNTHASE SUBUNIT F |
| Prupe.8G157200* | Protein kinase domain (Pkinase)//Leucine-Rich Repeat (LRR_1)//Leucine-rich repeat N-terminal domain (LRRNT_2)//Leucine-rich repeat (LRR_8) |
| Prupe.1G145400 | HISTIDINE-CONTAINING PHOSPHOTRANSFER PROTEIN 1 |

The genes marked with "*" were screened and subsequently tested for their ability to interact with *PpMYB52*.
